# Machine Learning-Driven Prediction of Composite Materials Properties Based on Experimental Testing Data

**DOI:** 10.3390/polym17050694

**Published:** 2025-03-05

**Authors:** Khrystyna Berladir, Katarzyna Antosz, Vitalii Ivanov, Zuzana Mitaľová

**Affiliations:** 1 Department of Applied Materials Science and Technology of Constructional Materials, Faculty of Technical Systems and Energy Efficient Technologies, Sumy State University, 116, Kharkivska St., 40007 Sumy, Ukraine; 2 Department of Automobile and Manufacturing Technologies, Faculty of Manufacturing Technologies, Technical University of Košice, Bayerova 1, 08001 Prešov, Slovakia; ivanov@tmvi.sumdu.edu.ua (V.I.); zuzana.mitalova@tuke.sk (Z.M.); 3 Faculty of Mechanical Engineering and Aeronautics, Rzeszow University of Technology, 35-959 Rzeszow, Poland; katarzyna.antosz@prz.edu.pl; 4 Department of Manufacturing Engineering, Machines and Tools, Faculty of Technical Systems and Energy Efficient Technologies, Sumy State University, 116, Kharkivska St., 40007 Sumy, Ukraine

**Keywords:** machine learning, prediction model, polymer composites, material optimization, process innovation, industry growth

## Abstract

The growing demand for high-performance and cost-effective composite materials necessitates advanced computational approaches for optimizing their composition and properties. This study aimed at the application of machine learning for the prediction and optimization of the functional properties of composites based on a thermoplastic matrix with various fillers (two types of fibrous, four types of dispersed, and two types of nano-dispersed fillers). The experimental methods involved material production through powder metallurgy, further microstructural analysis, and mechanical and tribological testing. The microstructural analysis revealed distinct structural modifications and interfacial interactions influencing their functional properties. The key findings indicate that optimal filler selection can significantly enhance wear resistance while maintaining adequate mechanical strength. Carbon fibers at 20 wt. % significantly improved wear resistance (by 17–25 times) while reducing tensile strength and elongation. Basalt fibers at 10 wt. % provided an effective balance between reinforcement and wear resistance (by 11–16 times). Kaolin at 2 wt. % greatly enhanced wear resistance (by 45–57 times) with moderate strength reduction. Coke at 20 wt. % maximized wear resistance (by 9−15 times) while maintaining acceptable mechanical properties. Graphite at 10 wt. % ensured a balance between strength and wear, as higher concentrations drastically decreased mechanical properties. Sodium chloride at 5 wt. % offered moderate wear resistance improvement (by 3–4 times) with minimal impact on strength. Titanium dioxide at 3 wt. % enhanced wear resistance (by 11–12.5 times) while slightly reducing tensile strength. Ultra-dispersed PTFE at 1 wt. % optimized both strength and wear properties. The work analyzed in detail the effect of PTFE content and filler content on composite properties based on machine learning-driven prediction. Regression models demonstrated high R-squared values (0.74 for density, 0.67 for tensile strength, 0.80 for relative elongation, and 0.79 for wear intensity), explaining up to 80% of the variability in composite properties. Despite its efficiency, the limitations include potential multicollinearity, a lack of consideration of external factors, and the need for further validation under real-world conditions. Thus, the machine learning approach reduces the need for extensive experimental testing, minimizing material waste and production costs, contributing to SDG 9. This study highlights the potential use of machine learning in polymer composite design, offering a data-driven framework for the rational choice of fillers, thereby contributing to sustainable industrial practices.

## 1. Introduction

Currently, the manufacturing of products from polymer composite materials (PCMs) is steadily growing in popularity, as these materials allow a significant improvement in the quality and weight reduction of products and structures [1], including those operating in extreme conditions [2], while at the same time increasing their reliability and resources [3].

Thus, based on a statistical data report [4], the advanced polymer composites market was valued at more than USD 10.5 billion in 2021, and is estimated to grow at a CAGR of around 5.9% from 2022 to 2030.

One of the most important conditions for PCMs’ competitiveness is the optimal combination of their manufacturability in production and application with highly functional characteristics (strength, stiffness, wear resistance) [5] and low cost [6]. The success of using chemistry in synthesizing new polymer materials [7] allowed them to be widely used in various branches of mechanical engineering [8,9,10]. The further expansion of their use is hindered by the lack of awareness among scientists and engineers as regards realizing their maximum potential in terms of their strength, reliability, and durability. These properties are particularly important because they are less studied than in traditional structural materials.

Composite materials are heterogeneous multi-component systems differing in chemical composition and properties. They are separated within the material by a well-defined boundary; some components are reinforcing components, and others are connecting matrices [9]. The main purpose of the matrix is to bind the filler together, to ensure the cooperation of all fibers (or dispersed particles), and to ensure the monolithicity of the material and the transmission (distribution) of stress [10]. The properties of the matrix almost completely depend on heat resistance, resistance to the effects of various working environments (water, steam, fuel, oil, etc.), impact viscosity, impact strength, resistance to long-term exposure to alternating loads, creep, and stress relaxation. The main purpose of the filler is to reinforce—that is, to strengthen—the matrix, to give the material the necessary special properties, and to reduce the cost of the part [11]. Fillers for composites are classified [12,13] by origin (organic, inorganic, synthetic), particle shape (fibrous, dispersed, nano-containing), and functional purpose (reinforcement, modification of properties, cost reduction). The main characteristics of fillers are their chemical composition, shape, particle size, density, mechanical strength, and ability to adhere to the polymer matrix [14]. The regularities of the influence of fillers on the mechanical properties of polymer composites are determined by their type, concentration, and distribution in the matrix. For example, fibrous fillers increase strength and wear resistance [15], dispersed fillers change stiffness and crack resistance and improve frictional properties [16], and nanofillers improve structural uniformity [17]. All this determines their high efficiency of use. Thus, to achieve a direct change in the properties of composite materials, it is necessary to consider many factors that affect their morphological features, namely, the chemical nature of the polymer matrix and filler, the phase state of the polymer, the adhesion of the polymer to the surface of the filler, the conditions for the formation of the filled polymer from a solution or melt, etc. [18].

Although the development of various industries requires the creation of highly efficient composite materials, the current economic landscape compels industries to prioritize composite materials that are both high-performing and cost-effective, driving innovation and sustainable practices within the sector. This reasoning justified the choice of components for the design of PCMs presented in this study. In addition, the proposed fillers have a different chemical nature, shape and size of particles, and a wide range of concentrations, which will allow for studying their influence on the functional characteristics of materials.

The compositions were made using polytetrafluoroethylene (PTFE) as a polymer matrix. Two types of fibers were chosen for research: carbon and basalt. Carbon fibers are a traditional, effective reinforcing filler used for polymer matrices [19,20,21,22,23]. The main disadvantage of carbon fibers, which limit their comprehensive use for the manufacturing of composites, is the high cost of the material. This reason necessitated the search for alternative, cheaper materials. Although basalt fibers are inferior to carbon fibers in reinforcement efficiency, they also possess properties necessary for filling PTFE. From an economic point of view, they are cheaper materials, as natural basalt is not a scarce and cheap raw material. The prospects of using basalt fibers as reinforcing fillers for PCMs were shown in [24,25,26,27,28]. Traditionally, solid lubricants such as graphite and coke are used for dispersed the filling of fluoropolymers, significantly increasing the wear resistance of pure PTFE [29,30,31]. This reason, as well as the availability and relative cheapness of the material, determined their choice as dispersed fillers for PTFE. Kaolin is a natural substance, and belongs to the group of silicates that make up the basis of the Earth’s crust and have an almost unlimited raw material base. The advantages of using this filler are the significant industrial reserves in the territory of Ukraine, its relative ease of extraction, and its affordable price, which make it a competitive filler for PCMs [32,33,34,35,36]. Ultra-dispersed PTFE was used as a nanosized filler. Significantly different in properties from industrial PTFE, ultra-dispersed PTFE has high thermodynamic compatibility with it, ensuring their effective interaction [37,38,39,40]. Two atypical fillers for PTFE were also studied—titanium dioxide nanofiller and sodium chloride. The analysis of the results of experimental studies [41,42,43,44] regarding the prospects of using nanosized titanium dioxide to strengthen polymer matrices necessitated the conducting of research on the PTFE matrix. Literary data on using sodium chloride as a dispersive filler were not found, so its effect will be tested experimentally in this work for the first time.

However, the rational choice of fillers and their sufficient concentration to give the matrix the necessary properties are extremely difficult problems. The optimal combination of fillers will provide a balance between the strength, ductility, and operational durability of the material. One of the options for its solution can be found in the application of computational methods to create a new scientific and methodical approach to forecasting the composition and properties of composite systems.

## 2. Computational Methods Used in Materials Science

**Figure 1:**
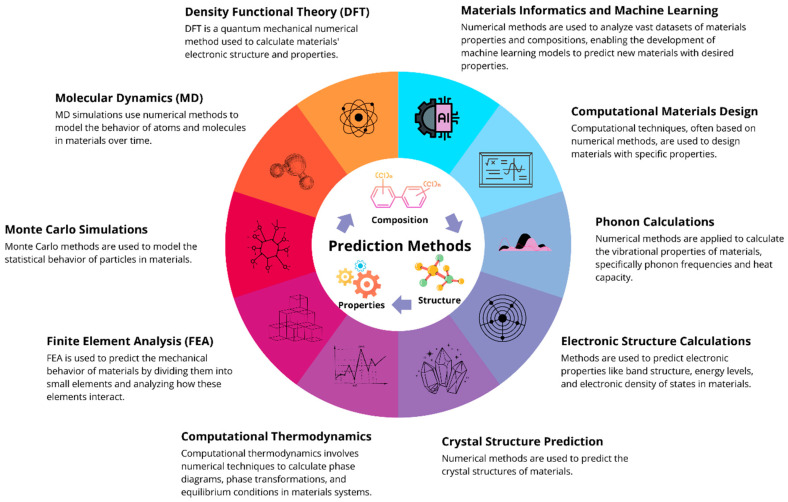
Commonly used types of computational methods for solving materials science tasks.

Computational methods are crucial in materials science in the context of determining material composition, predicting properties, and solving various problems. They can help to gain deep insights into the composition, structure, and properties of materials, and they play a significant role in developing new materials with tailored properties for various applications in engineering, electronics, energy, etc. [45]. Based on the literature review, we found ten main prediction methods used in materials science (Figure 1).

Density functional theory is a quantum mechanical approach used to determine electronic structures, band gaps, and magnetic properties in materials [46,47]. Molecular dynamics simulations model atomic and molecular behavior over time to predict thermal conductivity, mechanical properties, and phase transitions [48,49,50]. Monte Carlo simulations analyze statistical behavior, helping us to understand phase equilibria, diffusion, and defect formation [51,52]. Finite element analysis divides materials into small elements to assess mechanical behavior, stress, strain, and deformation [53,54,55,56,57]. Computational thermodynamics calculates phase diagrams and equilibrium conditions, particularly for alloys [58,59]. Crystal structure prediction methods aid in discovering new materials like superconductors and advanced polymers [60,61]. Electronic structure calculations, including Hartree–Fock and perturbation theory, predict band structures and energy levels [62,63]. Phonon calculations help determine vibrational properties, thermal conductivity, and expansion [64,65]. Computational materials design uses simulations to develop materials with improved strength, conductivity, and stability [66]. These methods collectively enhance material discovery and industrial applications [67,68].

Finally, materials informatics [69,70,71] and machine learning (ML) are used to analyze vast datasets of materials’ properties and compositions [72], enabling the development of prediction models for new materials with desired properties [73,74]. For example, leveraging data-driven strategies in material discovery [75] and process optimization can significantly enhance efficiency and reduce costs by streamlining workflows and improving the speed of material identification and optimization processes [76]. Thus, Yasniy O. et al. [77] used two types of ML for the simulation of the thermal conductivity coefficient of reinforced epoxy polymers. Despite obtaining different prediction errors, neural networks and boosted trees proved to be efficient in predicting, which makes them very promising for use in materials science. Combining ML and high-throughput computing can significantly enhance sustainability by optimizing resource utilization and minimizing experimental redundancies [78]. The ML approach has been actively applied in the industry sector, providing automation, accurate forecasting, and efficient resource management. Yadav D.K. et al. [79] focused on using ML algorithms in classifying machine failures and assessing the effectiveness of deep learning techniques for improved prediction accuracy with unbalanced datasets. Oladapo B.I. et al. [80] discovered that ML can improve the performance of renewable energy systems, resulting in a 15% enhancement in grid efficiency following optimization and a 10–20% boost in battery storage efficiency. Studies have convincingly proven the effectiveness of using ML to automatically detect manufacturing defects [81] and control plastic consumables [82], which increases the accuracy of quality control and reduces inspection and material costs. The reviews [83,84] summed up the possibilities of using ML to predict demand, optimize delivery routes, and reduce delays, which reduces logistics costs by up to 10–15% and increases demand forecast accuracy by up to 20–30%.

Based on the forward-looking experience in the application of an ML-driven approach for industrial application, this work is focused on using a machine learning algorithm to predict the properties and composition of PCMs based on a thermoplastic polymer matrix. Thus, this approach will optimize composite formulations by the rational selection of fillers for the polymer matrix, thereby reducing time and cost for experimental works, reducing material waste, and enhancing production efficiency, which contributes to SDG 9 (Industry, Innovation, and Infrastructure).

The scientific novelty of this study is based in developing a new methodology approach for the rational selection of fillers and predicting the functional properties of PCMs based on machine learning. For this purpose, it was necessary to solve the following research objectives. Firstly, the choice of fillers for the design of PCMs was substantiated, and the compositions of materials for experimental studies were developed, considering their effective concentrations. Secondly, the microstructure of the designed materials was investigated, and experimental tests were conducted to determine their functional properties. Thirdly, problems regarding predicting the properties or compositions of PCMs were solved using the machine learning algorithm. Finally, the verification of the results obtained from the research and calculations was carried out, based on which appropriate conclusions were made.

## 3. Materials and Methods

### 3.1. Materials and Their Properties

The main characteristics of PTFE [85] are presented in Table 1.

Shredded fibers obtained from unidirectional carbon fabric Carbon U-150 (Esslingen, Germany) were used in the work. Its chemical composition is given in Table 2.

Basalt ultrafine fiber produced by the Magma Industry (Kostopil, Ukraine) was used. Its chemical composition is given in Table 3.

Kaolin of KS-1 grade from the Prosyansk deposit (Prosyana, Ukraine) was used in the work. Its chemical composition is given in Table 4.

Ultra-dispersed PTFE brand «Forum» (trademark No. 140123), titanium dioxide brand SumTITAN R-2061 (PJSC “Sumikhimprom”, Sumy, Ukraine), and sodium chloride—food-grade rock salt extra ground (SE “Artemsil”, Soledar, Ukraine) (DSTU 3583:2015) were used in the work. The main characteristics of all used fillers are given in Table 5.

**Figure 2:**
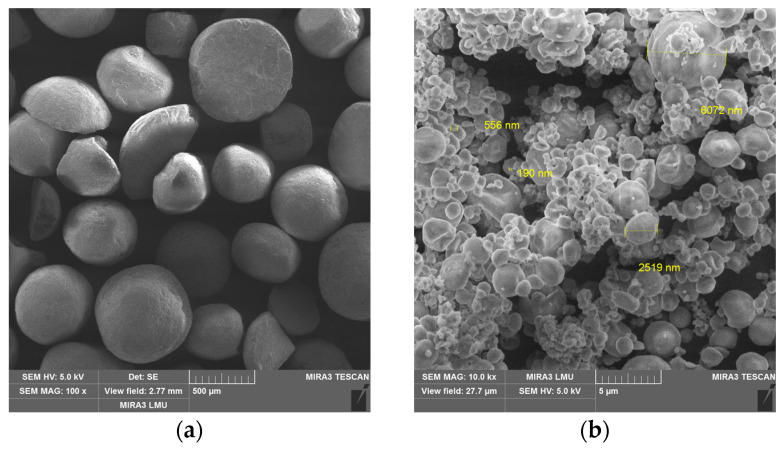

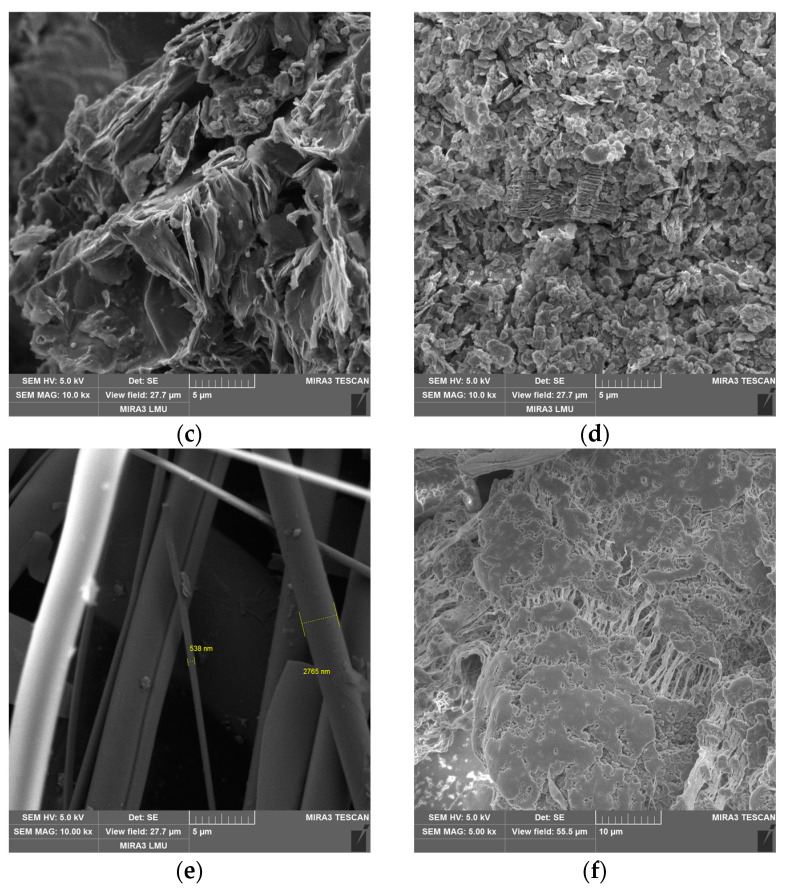
The microstructures of components used for designing PCMs: (**a**) sodium chloride; (**b**) ultra-PTFE; (**c**) graphite; (**d**) kaolin; (**e**) basalt fiber; (**f**) PTFE (matrix).

The microstructures of some of the above components used for creating PCMs are shown in Figure 2.

### 3.2. Experimental Materials Design

The concentrations chosen for the selected fillers are based on previous studies by various authors [19,20,21,22,23,24,25,26,27,28,29,30,31,32,33,34,35,36,37,38,39,40,41,42,43,44]. The concentrations of carbon fillers (carbon fibers, coke, graphite) varied in a wide range from 5 to 25 wt. %; the concentration of basalt fiber was close to that of carbon fibers (for comparison); that of kaolin ranged from 2 to 6 wt. %, sodium chloride from 2 to 8 wt. %, and nanofillers (ultra-PTFE, titanium dioxide) from 1 to 5 wt. %.

Two-component (Table 6) and three-component systems (Table 7) of PCMs were developed and investigated in the work.

### 3.3. Production of Test Samples

The weakest place in the structure of a filled polymer system is the boundary layer between the matrix and the fillers, since the material’s destruction occurs along the interphase boundaries. Therefore, to improve the efficiency of the interaction between PTFE and fillers, it is necessary to improve the operation of the mixing of initial components.

**Figure 3:**
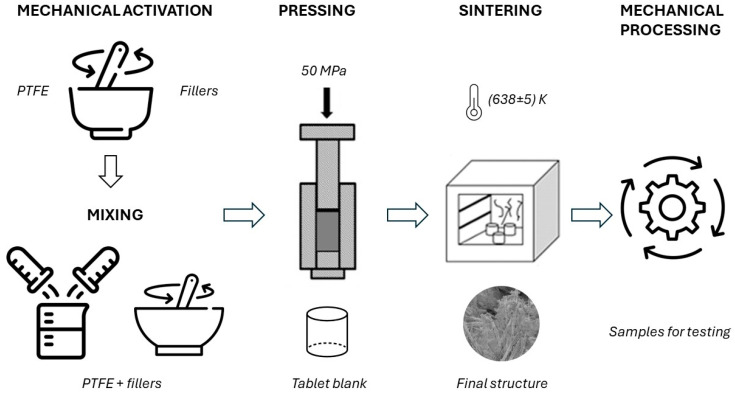
Flowchart of the production process for obtaining test samples.

Samples for these experimental tests were obtained by traditional methods of powder metallurgy (pressing and sintering) with the additional mechanical activation of components before and during the mixing process (Figure 3). An increase in the surface energy of both the PTFE particles and the filler occurs during their activation, which is accompanied by an increase in the adhesion of the filler particles to the polymer. This, in turn, leads to the further crystallization of the polymer, leading to the formation of a structure with a relatively high packing density of structural elements, and their orderliness.

### 3.4. Materials Testing

The testing of designed composites included determining density, strength, relative elongation, and wear intensity as the main and necessary data regarding the polymer-based material designed for industrial use. The testing procedure of samples was carried out based on the technical conditions TC U 22.2–33729459–001:2014 Blanks for fluoroplastic compositions (FC) (Ukraine).

The density of materials was determined by the method of hydrostatic weighing. The masses of equal volumes of the substance under study and a liquid of known density (distilled water) were compared by weighing twice—first in air and then in water. The accuracy of density measurement was up to 0.1%. Weighing was carried out at room temperature on a VLA-200-M scale with an accuracy of 2 mg.

Tensile strength and relative elongation were determined for ring samples using hard half-discs on the MP-05-1 tensile testing machine. This machine has a maximum test force of 500 kgf and operates with a 0.27 kW motor at 1400 rpm. Samples in the form of a ring with a radial thickness of 2 ± 0.2 mm and an axial height of 6 ± 1 mm were cut from a workpiece with an outer diameter of 50 ± 5 mm and an inner diameter of 30 ± 5 mm. Radial thickness and axial height were measured at four diametrically opposite points. The test was carried out at a temperature of 23.0 ± 2.0 °C with a speed of movement of the cutting machine grip 100 ± 10 mm/min.

The tensile strength (σ), MPa, was calculated by the formula

(1) $$\sigma =\frac{P}{2hh_{1}}=\frac{P}{2S},$$

where P is the breaking force, N; h is the radial wall thickness of the ring specimen, m; h_1_ is the axial height of the ring specimen, m; S is the minimum cross-section of the ring specimen, m^2^.

The relative elongation (δ), %, was calculated with the formula

(2) $$\delta =\frac{∆l}{l_{0}}\cdot 100\%,$$

where ∆l is the change in the calculated length of the sample at the moment of rupture, mm; l_0_ is the initial calculated length of the sample, mm.

The test result was taken as the arithmetic mean of three determinations, the difference between the most different values of which should not exceed the permissible difference, which is 15% when determining strength and 30% when determining relative elongation at break from the calculated arithmetic mean value.

The intensity of wear was determined on a serial friction machine 2070 CMT–1 according to the “partial insert-shaft” scheme without external lubrication. This equipment operates at a rotational speed of 75–1500 rpm, with a maximum friction torque of 20 N·m and load ranges of 200–2000 N and 500–5000 N. The counter-body was a ø48 mm roller made of AISI 1045 steel (HRC 45, Ra 0.72 μm). The partial insert was made of the material under study and was a sector with a width of 16 mm from a ring ø80 by ø60 mm with a height of 9 mm. The run-in and testing for each material sample were carried out one track at a time. The amount of wear of the samples was determined gravimetrically on analytical scales with an accuracy of 10^−5^ g and was converted into wear intensity by the formula

(3) $$I=\frac{V}{P\cdot S}.$$

where V is the volume of worn material, mm^3^; P is the normal load, N; S is the friction path, m.

The relative linear velocity and specific load of the friction pair were selected in accordance with the real friction pairs “piston rings—cylinder” in the compressor 4GM 2.5 U–3.4/2.8–251 (pressure P = 12 MPa, linear velocity 2.0 m/s). The root means square error in assessing the intensity of PCMs wear was regulated by the errors in measuring the sample mass, speed, and duration of friction, and did not exceed 5%.

The microstructure of composite samples was studied using a high-resolution field emission scanning electron microscope TESCAN MIRA 3 LMU (TESCAN GROUP, a.s., Brno, Czech Republic) with a Schottky emitter, capable of operating at accelerating voltages from 200 V to 30 kV, providing high-magnification imaging in low-vacuum mode for non-conductive materials to allow detailed microstructural and compositional analysis in materials testing.

### 3.5. Machine Learning Approach

The linear regression model is one of the simplest and most commonly used methods in statistical analysis and machine learning. Its main goal is to find the relationship between a dependent variable (y) and one or more independent variables (x). The linear regression equation for one independent variable (simple linear regression) can be written as:

y = β0 + β1x + ε,(4)

where y is the dependent variable, x is the independent variable, β0 is the intercept (the point where the line crosses the y-axis), β1 is the slope coefficient, and ε is the error term.

For multiple independent variables (multiple linear regression), the equation is:

y = β0 + β1x_1_ + β2x_2_ + ... + βpx_p_ + ε, (5)

The most commonly used method for estimating the coefficients β0 and β1 (and other βp in the case of multiple regression) is the method of least squares (OLS—Ordinary Least Squares). This method minimizes the sum of the squares of the differences between the actual values and the values predicted by the model.

The assumptions of the linear regression model include the following:

- Linearity (the relationship between the dependent variable and the independent variables is linear);
- Independence (observations are independent of each other);
- Homoscedasticity (constant variance of the errors);
- Normality of the errors (errors are normally distributed for statistical tests).

The quality of the model fit can be assessed using several measures, the most popular of which are R^2^ (coefficient of determination), which measures the proportion of the variability in the dependent variable that is explained by the independent variables, and residual analysis, which helps to check whether the assumptions of the model are met (e.g., normality, homoscedasticity).

## 4. Experimental Results

### 4.1. Microstructure Analysis

**Figure 4:**
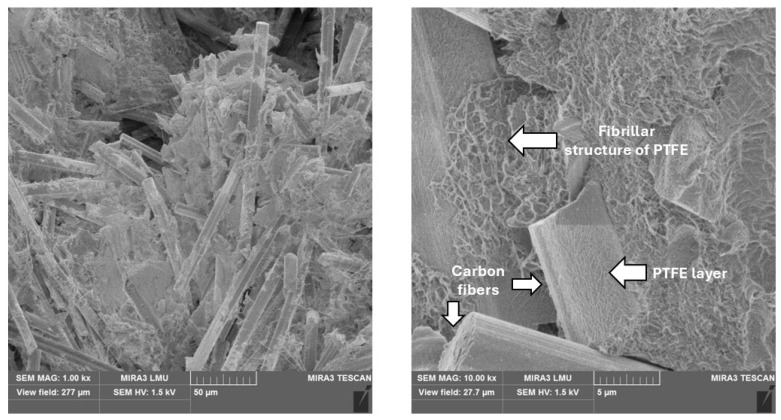
The microstructure of the composite with 20% carbon fibers.

PTFE + carbon fibers. As shown in Figure 4, the initial structure of the polymer (Figure 2f) with long lamellar formations is broken into smaller sections by carbon fibers. Areas of polymer with a structure of PTFE appear in the matrix, which can be identified as fibrillar (filamentous). A clearly defined layer of polymer is observed on the surfaces of carbon fibers, indicating a high-quality process of mixing the composite components and a high level of adhesion between them. A homogeneous composite structure is formed with decreased voids and a more uniform distribution of fibers in the matrix. Surface-modified fibers with a layer of PTFE in contact with the matrix form a secondary form of interaction as physical adhesion based on weak intermolecular van der Waals forces. This leads to reducing the defectiveness of the composition and the probability of defects during the formation of the composite.

**Figure 5:**
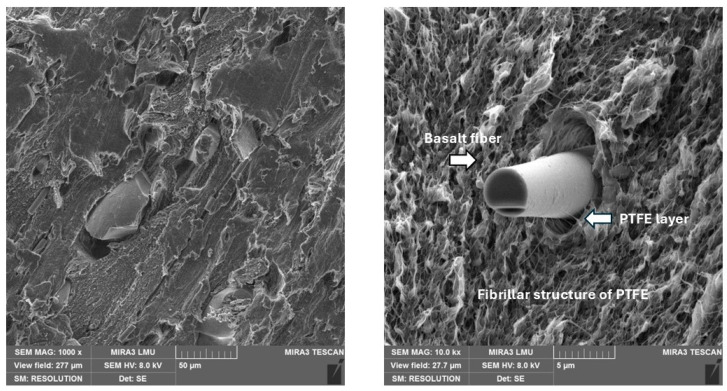
The microstructure of composite with 10% basalt fibers.

PTFE + basalt fibers. When filling PTFE with basalt fibers, a fibrillar structure of the polymer was also observed (Figure 5). This contributed to increasing the adhesive interaction of the components of the composition, creating a layer of polymer on the fiber surface. It is visually noticeable that the thickness of the formed layer on the surface of basalt fiber is smaller than when using carbon fiber. This thesis was confirmed by the further analysis of the results of mechanical tests of composites. Low indicators of mechanical properties of composites with a filling of more than 10% are explained by the loosening of the volume of the material. The effect of basalt fibers on the polymer is to increase the density of the structure due to the formation of interphase layers with a special stacking of PTFE macromolecules at the interface between the matrix and the filler.

**Figure 6:**
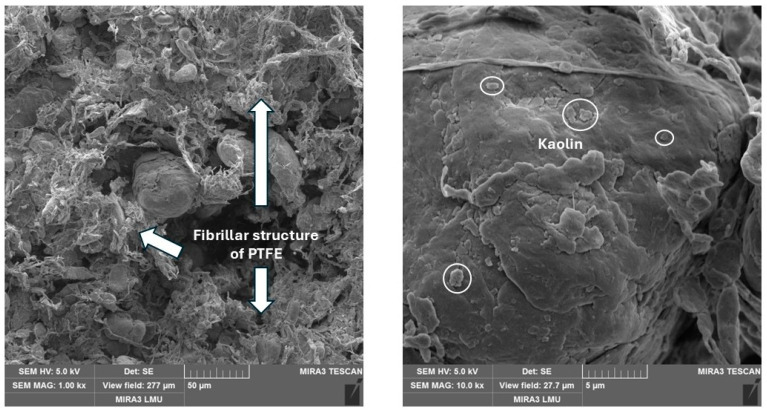
The microstructure of the composite with 2% kaolin.

PTFE + kaolin. When filling the polymer matrix with kaolin, the effect of the oriented action of the filler is manifested; i.e., due to kaolin, macromolecules of non-polar PTFE are intercalated in the space between the plates of the filler with an increased value of the uncompensated charge. The kaolin surface appears partially embedded in the polymer space, indicating the interaction of the two elements at the interface (Figure 6). The fibrillar structure of PTFE was observed at a low kaolin content, which contributes to the effective lubrication of surfaces during friction, which was confirmed by a significant reduction in wear intensity. An increase in the concentration of the filler also leads to the aggregation of kaolin particles.

**Figure 7:**
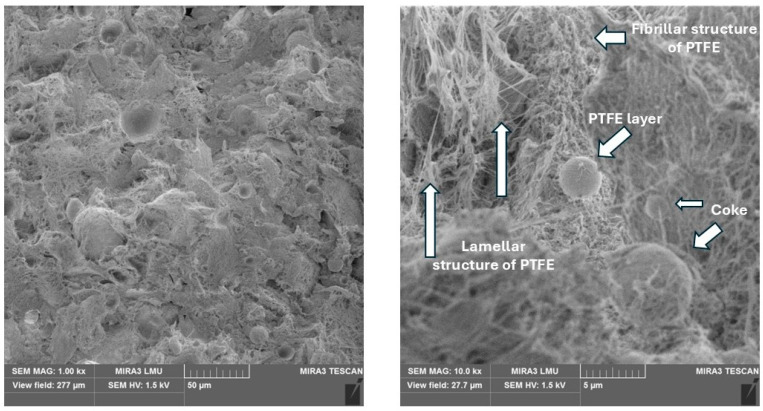
The microstructure of the composite with 20% coke.

PTFE + coke. The introduction of coke contributes to the formation of the fibrillar structure of PTFE composites. Together with the lamellar structure’s characteristic of unfilled PTFE, spherical coke particles are identified (Figure 7). Coke has a developed porous surface that can form a physical adhesion with the polymer matrix; a dense layer of polymer on the coke surface was visually observed. This improves the self-lubricating properties of the composite under unlubricated friction conditions, which correlates with friction test results.

**Figure 8:**
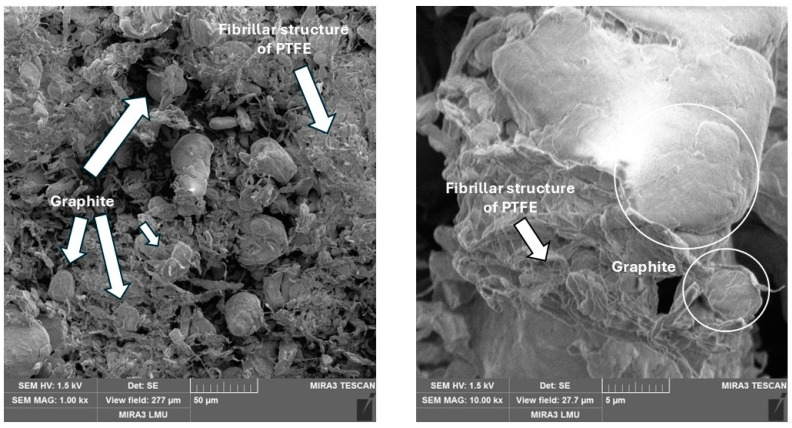
The microstructure of composite with 10% coke.

PTFE + graphite. The addition of graphite, which is similar to kaolin in chemical nature and particle shape when interacting with PTFE, contributes to the formation of a fibrillar structure (Figure 8), which improves wear intensity without a loss of mechanical strength. However, some loosening of the structure of the material is also observed, which is obviously due to the insufficient quality of mixing of the original ingredients.

**Figure 9:**
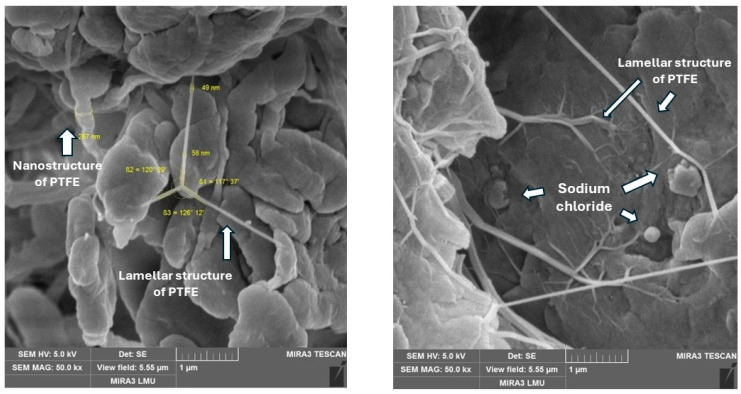
The microstructure of the composite with 2% sodium chloride.

PTFE + sodium chloride. An interesting structure of the composite was recorded with the addition of sodium chloride. Along with the usual elements typical for PTFE, the formation of nano-sized formations of the polymer matrix and lamellar strands with a thickness of 50 nm is observed, which form a regular three-rayed star (Figure 9, left). Particles of sodium chloride are randomly arranged within the material, contacting such polymer lamellae (Figure 9, right). At the same time, the formation of a polymer layer on the surface of the sodium chloride particle is not noticeable, as in the case of coke. This indicates an unstable contact of the filler with the matrix and an inactive surface of the filler. This thesis is confirmed by the results of mechanical and tribological tests.

**Figure 10:**
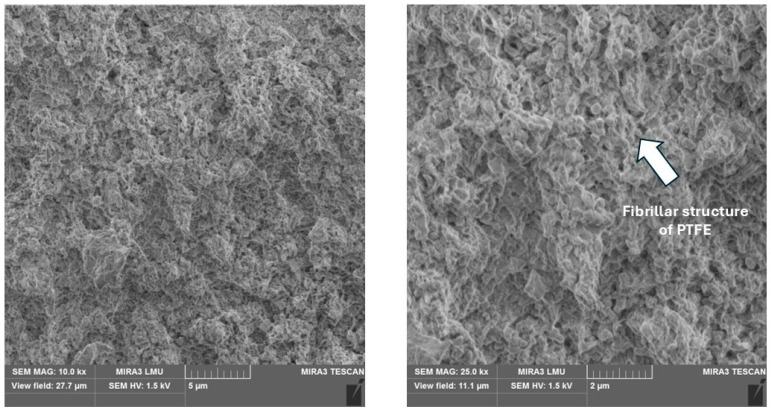
The microstructure of composite with 5% titanium dioxide.

PTFE + titanium dioxide. Nano-sized titanium dioxide, having a large surface area, is a chemically active filler that more easily interacts with the polymer matrix. In Figure 10, the fibrillar structure of the polymer with good polymer–nanoparticle interaction is observed. This contributes to a good dispersion and distribution of nanoparticles in the polymer matrix. A feature of composites with nanofillers is their small size, which leads to an increase in the interfacial area of the material compared to larger fillers. Achieving a structure with a large interfacial area is an effective strategy for improving the mechanical properties of composites, which is consistent with the results of strength tests.

**Figure 11:**
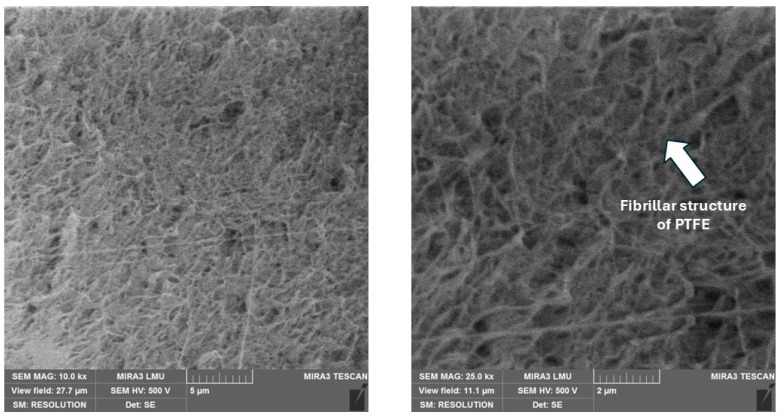
The microstructure of composite with 1% ultra-PTFE.

PTFE + ultra-PTFE. The introduction of nanosized ultra-PTFE in small amounts (up to 5 wt. %) into the polymer matrix leads to the formation of a supramolecular fibrillar structure with an increased material density (Figure 11). Due to the unique microstructure of ultra-PTFE, which was described in detail in [39], there is increased activity during interaction with the main polymer and the formation of a transferred layer during frictional contact. This, in turn, reduces the defectivity of the interphase boundary in the polymer composite, and contributes to the improvement of the wear resistance of the composite. This statement is confirmed by the data [38,40].

### 4.2. Testing Analysis

**Figure 12:**
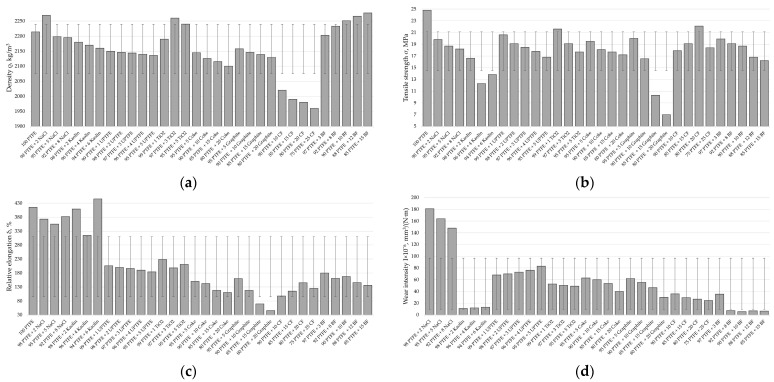
The functional properties of the designed two-component PCMs based on the filler concentrations: (**a**) density, (**b**) tensile strength, (**c**) relative elongation, (**d**) wear intensity (for 100% PTFE, the wear intensity is 610 × 10^−6^ mm^3^/N·m).

The functional properties of the designed two-component PCMs are presented in Figure 12.

Having analyzed the data in Figure 12 and comparing the values of density, tensile strength, relative elongation, and wear intensity depending on the concentration range for each of the fillers, it is possible to draw conclusions about the optimal content of the filler.

When adding carbon fibers to the polymer matrix, depending on the concentration of the latter, it reduces the level of tensile strength by 12–39% and relative elongation by 2.9–4.2 times, with a significant increase in wear resistance by 17–25 times. Therefore, the optimal concentration of the carbon filler is 20 wt. %, which corresponds to the formation of a more homogeneous structure of the composite, thus providing sufficient functional properties.

Basalt fibers, depending on their concentration, reduce the tensile strength of pure PTFE by 28–54% and relative elongation by 2.5–4.3 times, while increasing wear resistance by 11–16 times. The optimal set of properties of the composite is achieved with a content of 10 wt. % basalt fibers.

Depending on the concentration of the kaolin in the PTFE matrix, it reduces the latter’s tensile strength by 1.5–2.3 times and its relative elongation by 1.5–42%, with a significant increase in wear resistance by 45–57 times. The optimal set of properties of the composite is achieved with a content of 2 wt. % kaolin.

The optimal concentration of coke in the composite is 20 wt. %, which provides the maximum value of wear resistance at the required level of mechanical properties. The addition of coke to the polymer, depending on the concentration of the latter, reduces its level of breaking strength by 27–44% and relative elongation by 2.8–3.7 times, while increasing wear resistance by 9–15 times.

With an increase in the graphite content to 15 wt. %, the mechanical properties of the composition decrease sharply—tensile strength by 63.5% and relative elongation by 69%—while wear resistance increases linearly. A further increase in the concentration of graphite to 20 wt. % leads to a decrease in mechanical properties by almost 2 times and wear intensity by 30%. Therefore, to achieve optimal operational properties of the PTFE composite, the concentration of graphite should not exceed 10 wt. %.

The addition of sodium chloride to the PTFE matrix, depending on the concentration of the latter, reduces its level of tensile strength by 25–36% and relative elongation by 8–17%, with a moderate increase in wear resistance by 3–4 times. The optimal set of properties of the composite is achieved with a content of 5 wt. % sodium chloride.

Titanium dioxide, depending on its concentration, reduces the level of strength of unfilled polymer by 15–40% and its relative elongation by 1.8–2.1 times, while increasing wear resistance by 11–12.5 times. The optimal set of properties of the composite is achieved with a content of 3 wt. % TiO_2_.

Filling the polymer with nano-sized UPTFE reduces its tensile strength by 20–48% and increases wear resistance by 7–9 times. With an increase in the filler content from 1 to 5 wt. %, the tensile strength of the composite decreases by 22.6%, and wear increases by 23.0%. Therefore, the addition of 1 wt. % UPTFE provides maximum strength and wear-resistant parameters.

**Figure 13:**
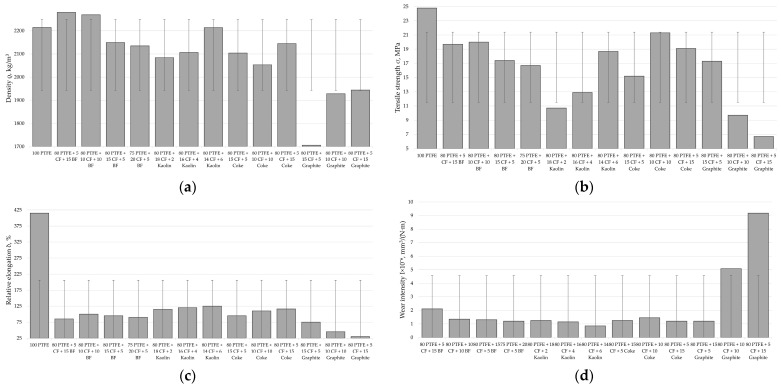
The functional properties of the designed three-component PCMs based on the filler concentrations: (**a**) density, (**b**) tensile strength, (**c**) relative elongation, (**d**) wear intensity (for 100% PTFE, the wear intensity is 610 × 10^−6^ mm^3^/N·m).

The functional properties of the designed three-component PCMs are presented in Figure 13. The analysis of this figure shows that for three-component composites, the best performance indicators are observed with the following ratio of ingredients: 10% carbon and 10% basalt fibers; 14% carbon fibers and 6% kaolin; 5% carbon fibers and 15% coke; 15% carbon fibers and 5% graphite.

## 5. Prediction Results Analysis and Discussion

### 5.1. Effect of PTFE Content on Composite Properties

The results obtained during the research were subjected to a detailed analysis. Firstly, the changes in the values of density, tensile strength, relative elongation, and wear intensity depending on the main component of the PTFE composite were analyzed. The analysis included a thorough comparison of the results obtained for different variants of the composite to identify the influence of each component on the mechanical and physical properties studied. The results obtained are presented in the figures as box plots, illustrating the variation of each parameter as a function of the component variables.

**Figure 14:**
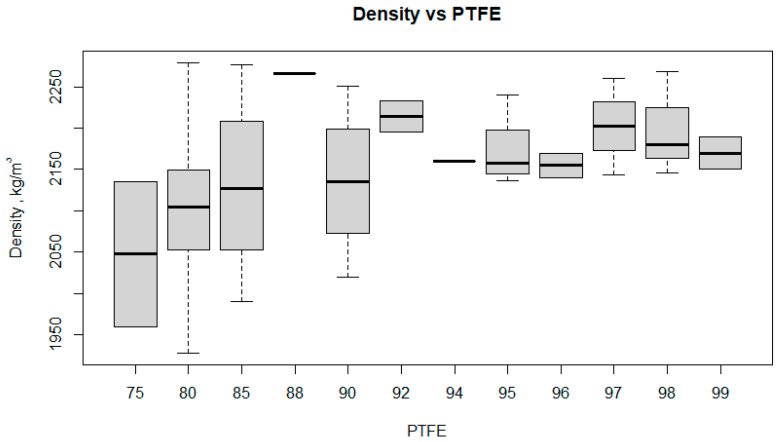
Boxplot density versus PTFE.

The presented box plot (Figure 14) illustrates the relationship between the density of the composites and the PTFE content.

For a lower PTFE content (75−85%), the density is more variable, ranging from approximately 1950 kg/m^3^ to 2250 kg/m^3^. With higher PTFE content (88−99%), the density’s variability is smaller, and the values are more consistent. The median density increases from around 2000 kg/m^3^ at 75% PTFE to about 2150 kg/m^3^ at 80% PTFE, then stabilizes. Greater variance occurs at lower PTFE content (75−85%), while at higher PTFE content (above 85%), the variance is smaller, indicating a more homogeneous composite composition. In some cases (e.g., 85% PTFE), outliers are present. The density changes in the composite are dependent on the PTFE content. A higher PTFE content (above 85%) leads to more stable density, suggesting better material homogeneity. Lower PTFE content (75−85%) causes greater density variability, which may affect composite heterogeneity. These observations are crucial in the development of PTFE composites, where the density is an informative parameter that determines the structure of the materials and correlates well with other parameters characterizing the quality of the composite (strength, wear resistance).

**Figure 15:**
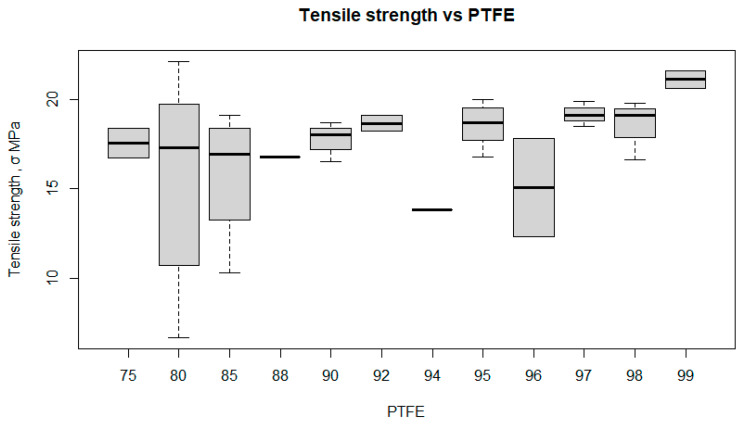
Box plot tensile strength versus PTFE.

The box plot (Figure 15) illustrates the relationship between the tensile strength (TS) of the composite and its PTFE content.

At 75% PTFE, the TS is relatively stable, with a median of around 15 MPa. At 80% PTFE, the tensile strength is more variable, ranging from about 10 MPa to 20 MPa, with a median of about 17 MPa. For 85% PTFE, the variability is also significant, with values ranging from about 10 MPa to 20 MPa, but with a lower median of about 14 MPa. For 88% PTFE, the TS is more stable, with a median of around 16 MPa and less variability. For 90% PTFE, the value is stable at around 16 MPa. The PTFE contents of 94% and 96% show little variability, but for 96% PTFE, the median is around 13 MPa, which is significantly lower than the other values. Tensile strength is higher and more stable for 97%, 98%, and 99% PTFE, with medians around 15 MPa, 17 MPa, and 20 MPa, respectively.

The relationship between TS and PTFE content is variable. The strength is more stable at PTFE contents of 75%, 88%, 90%, 94%, 97%, 98% and 99%, while the variability is greater at 80%, 85% and 96%. A higher PTFE content (99%) gives the highest TS, suggesting that increasing PTFE content can improve the mechanical properties of the composite, although, at certain values (such as 96%), there can be significant variability and lower median values.

This conclusion can be explained by the fact that due to the chemical inertness of PTFE macromolecules, no chemical bonds are formed at the interface with the filler, and due to the low surface energy and high viscosity, the good wetting of the filler surface by the melt is not ensured. As a result, the interphase layer is not capable of transmitting the applied load, and during tensile tests of composites, the reinforcing filler does not actually contribute to increasing the tensile strength of the sample. Therefore, the tensile strength value is an indicator of the quality of filled PTFE—unlike all other polymers, its filling with any component using traditional technologies leads to a decrease in the tensile strength of the composite. The degree of strength reduction is almost linearly dependent on the degree of filling, and does not depend on the shape of the particles. This corresponds with the results in [86,87].

**Figure 16:**
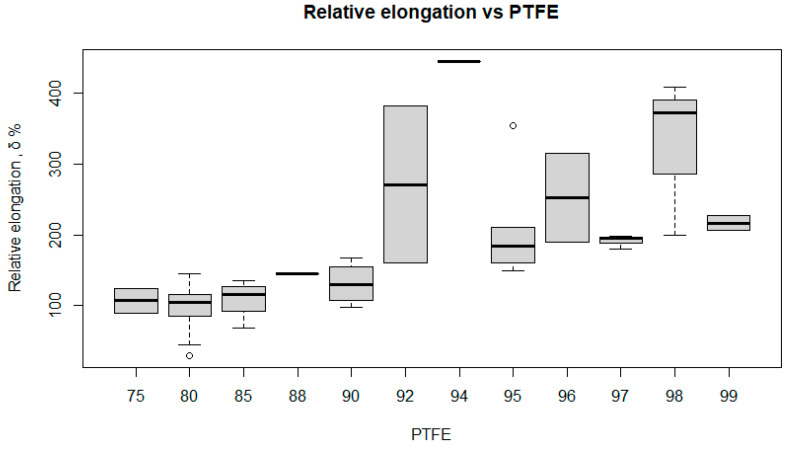
Box plot of relative elongation versus PTFE.

Figure 16 shows the relationship between the relative elongation (RL) of the composite and the PTFE content.

At 75% PTFE, the RL is relatively low and stable, with a median of around 120%. For 80% PTFE, the RL is slightly more variable, ranging from about 100% to 150%, but most results cluster around a median of about 120%. At 85% PTFE, the RL is similar, with a median of around 110% and some outliers. At 88% PTFE, the RL is more stable, with a median of around 110%. At 90% PTFE, the RL increases, with a median of around 150% and slight variability. At 92% PTFE, the RL increases significantly to a median of about 300%, but with a large variability ranging from about 200% to 400%. At 94% PTFE, the relative elongation is more stable, with a median of around 150%. At 96% PTFE, the RL is lower, with a median of around 100% and less variability. At 97% PTFE, the median RL is around 120%, but the variability is significant. At 98% PTFE, the RL increases significantly, with a median of around 200% and variability from around 100% to 300%. For 99% PTFE, the RL is lower and more stable, with a median of around 110%.

The RL of the composite is clearly dependent on the PTFE content. Lower PTFE contents (75–85%) result in stable but lower RL values. Higher PTFE contents (90–98%) show higher RL values but with greater variability, especially at 92% PTFE. The highest RL values are observed at 92% and 98% PTFE.

**Figure 17:**
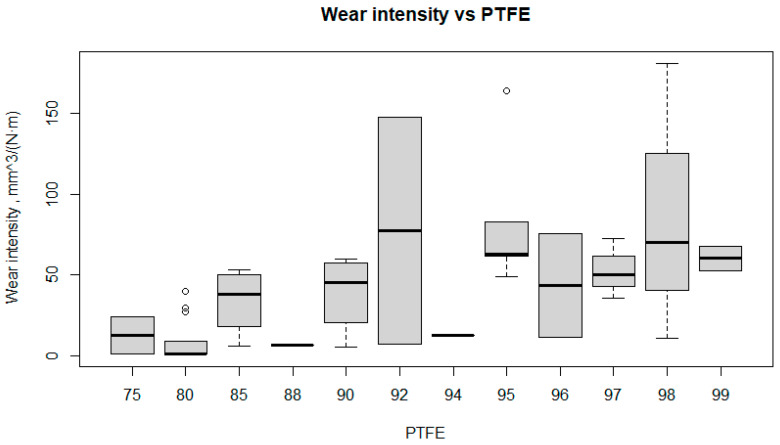
Box plot wear intensity versus PTFE.

When analyzing the results in Figure 17 above, it should be noted that at 75% PTFE, the wear intensity (WI) is relatively low and stable, with a median of around 30 mm^3^/(N·m). At 80% PTFE, the WI is also low, with a median of about 20 mm^3^/(N·m), but there are a few outliers. At 85% PTFE, the WI values are stable at around 25 mm^3^/(N·m).

At 90% PTFE, the WI increases slightly to a median of about 45 mm^3^/(N·m). At 92% PTFE, the WI increases significantly and reaches a median of about 100 mm^3^/(N·m), with a large variability from about 50 mm^3^/(N·m) to 150 mm^3^/(N·m). A PTFE content of 94% shows a low wear intensity with a median close to zero. At 95% PTFE, the WI is relatively low, with a median of about 30 mm^3^/(N·m). At 96% PTFE, the WI is stable, with a median of about 20 mm^3^/(N·m). For 97% PTFE, the WI is higher, with a median of about 50 mm^3^/(N·m) and greater variability. For 98% PTFE, the WI increases again, with a median of about 75 mm^3^/(N·m) and a large variability ranging from about 30 mm^3^/(N·m) to 150 mm^3^/(N·m). For 99% PTFE, the WI is stable, with a median of about 40 mm^3^/(N·m).

The WI of the composite is clearly dependent on the PTFE content. Lower PTFE contents (75–85%) result in low and stable wear intensity. Higher PTFE contents (90–99%) show greater variability, particularly at 92% and 98%, with the highest wear intensity. This fact closely correlates with the results of the experimental analysis discussed above, and of another study [33,34,88,89].

### 5.2. Effect of Filler Content on Composite Properties

The next stage of the analysis was to develop regression models for the properties of density, tensile strength, relative elongation, and wear intensity, considering not only the PTFE component but also the fillers used—coke, titanium dioxide, kaolin, ultra-PTFE, sodium chloride, graphite, carbon fiber, and basalt fiber. First, the effect of each of these components on the various mechanical and physical properties of the composite was analyzed.

For each property, separate regression models were created to identify the influence of each independent variable on the dependent variable. In the analysis of density, the change in the density of the composite was evaluated as a function of the proportions of the different components. Similarly, for tensile strength, the components that have the greatest influence on the strength of the material were examined. Relative elongation analyzed the elasticity of the composite, while wear intensity assessed the resistance of the material to wear under different conditions.

Preliminary regression models indicated that the basalt fiber variable was linearly dependent on other variables in the model, including PTFE, coke, titanium dioxide, kaolin, ultra-PTFE, sodium chloride, graphite, and carbon fiber. This means that the basalt fiber (BF) variable can be expressed as a linear combination of these variables. In practice, this means that the coefficient for BF cannot be estimated independently of the other variables, making the design matrix singular. This has the following consequences: the BF variable is fully dependent on the other variables, making it impossible to independently estimate its effect on the dependent variable; due to this full dependence, the regression model cannot estimate the coefficient for BF, leading to problems in interpreting the model.

The solution to this problem is to remove the BF variable from the model, allowing the coefficients for the remaining variables to be correctly estimated. Subsequent analyses used a variant wherein regression models were developed without the BF variable. In future studies, further analyses should also be carried out to diagnose the multicollinearity of the remaining variables to ensure that the problem of multicollinearity has been fully resolved.

When analyzing the results of the linear regression model, it should be noted that they reveal the relationship between the density of the composite and various independent variables, such as the contents of PTFE, coke, titanium dioxide, kaolin, ultra-PTFE, sodium chloride, graphite, and carbon fiber.

The linear regression model is as follows:

Density = 3266.922 − 11.139⋅PTFE − 12.681⋅Coke + 11.510⋅Titanium_dioxide + 1.376⋅Kaolin − 14.438⋅Ultra_PTFE − 2.617⋅Sodium_chloride − 15.750⋅Graphite − 17.685⋅Carbon_fiber (6)

The residuals of the model indicate the differences between the observed values and the values predicted by the model. These values are symmetrically distributed around a median close to zero, indicating a good fit of the model to the data. The minimum and maximum residuals are −113.464 and 98.925, respectively, with first and third quartiles of −24.200 and 33.242 and a median of −0.322.

The regression coefficients show the impact of each variable on the dependent variable. The intercept is 3266.922 and it is highly statistically significant (*p* < 0.001). The variables PTFE, coke, graphite, and carbon fiber have a significant influence on the dependent variable, with corresponding coefficients of −11.139, −12.681, −15.750, and −17.685 and very low *p*-values (*p* < 0.001). The variable ultra-PTFE has a coefficient of −14.438 and is close to significance (*p* = 0.0896). The variables titanium dioxide, kaolin, and sodium chloride do not significantly affect the dependent variable, suggesting that they may be less important in the context of this model, with *p*-values of 0.2416, 0.8047, and 0.6600, respectively. The results clearly indicate that the contents of PTFE, coke, graphite, and carbon fiber significantly affect the density of the composite and reduce its value. This conclusion is well correlated with experimental testing data (Figure 12a), showing that dispersed fillers (titanium dioxide, kaolin, and sodium chloride) with up to 10% content do not lead to a significant change in the density of the polymer matrix. Instead, for coke, graphite, and carbon fibers with up to 20−25% content, a clearly visible linear dependence of the decrease in the density of the polymer matrix depending on the increase in the filler concentration is observed. Therefore, the model has effectively determined the factors impacting the significance of the influence of fillers. Furthermore, the obtained R-squared value is 0.74, which means that the model explains 74% of the variability in the composite density. This is quite a high value, indicating the good quality of the model.

The linear regression model for tensile strength (TS) presents the relationship between the TS of the composite and various fillers, such as the content of PTFE, coke, titanium dioxide, kaolin, ultra-PTFE, sodium chloride, graphite, and carbon fiber.

The linear regression model is as follows:

Tensile strength = 5.81435 + 0.14369⋅PTFE + 0.02793⋅Coke − 0.26339⋅Titanium_dioxide − 0.89622⋅Kaolin − 0.46079⋅Ultra_PTFE − 0.11487⋅Sodium_chloride − 0.54474⋅Graphite + 0.04845⋅Carbon_fiber(7)

The residuals of the model indicate the differences between the observed values and the values predicted by the model. These values are symmetrically distributed around a median close to zero, indicating a good fit of the model to the data. The minimum and maximum residuals are −5.6890 and 6.0897, respectively, with first and third quartiles of −0.8927 and 0.5917 and a median of 0.0348.

The regression coefficients show the impact of each variable on the dependent variable. The intercept is 5.81435 and is not statistically significant (*p* = 0.56536). The PTFE variable has a coefficient of 0.14369, but its effect is insignificant (*p* = 0.18519). The variable coke has a coefficient of 0.02793, and is also not significant (*p* = 0.79185). The variable titanium dioxide has a coefficient of −0.26339, and is not significant (*p* = 0.55052). The variable kaolin has a coefficient of −0.89622, and is statistically significant (*p* = 0.00103). The variable ultra-PTFE has a coefficient of −0.46079, but is not significant (*p* = 0.22605). The variable sodium chloride has a coefficient of −0.11487, and is also not significant (*p* = 0.66945). The variable graphite has a coefficient of −0.54474 and is highly statistically significant (*p* < 0.001). The variable carbon fiber has a coefficient of 0.04845 and is not significant (*p* = 0.64321). The model shows that the variables kaolin and graphite significantly affect the composite’s TS, reducing its value. The variable PTFE, although it has a positive coefficient, is not statistically significant. This is explained by the findings of an analysis of the experimental test results (Figure 12b and Figure 13b)—all fillers lead to a decrease in the strength of the polymer matrix, which is typical for PTFE [22,23,32,86,87]. But it is kaolin and graphite that lead to a dramatic (in some places critical) drop in the strength of the composite in contrast to other fillers. Therefore, the model identified them as the most significant, although the influence of other components is also important.

The R-squared value for the model is 0.67, which means that the model explains 67% of the variability in the tensile strength of the composite. This relatively high value indicates a good fit of the model to the data. The model is well-fitted to the data, as indicated by the high R-squared value and the symmetric distribution of the residuals around the median. Nevertheless, for the correct interpretation of the strength prediction, it is necessary to consider additional analyses of the impact results, which indicate the insufficient accuracy of this model.

The next model presents the relationship between the relative elongation (RL) of the composite and various fillers, such as the contents of PTFE, coke, titanium dioxide, kaolin, ultra-PTFE, sodium chloride, graphite, and carbon fiber.

The linear regression model is as follows:

Relative elongation = −559.4071 + 8.0785⋅PTFE + 0.3735⋅Coke − 2.1771⋅Titanium_dioxide + 23.0490⋅Kaolin − 7.4229⋅Ultra_PTFE + 27.9755⋅Sodium_chloride − 3.5091⋅Graphite + 0.3001⋅Carbon_fiber(8)

The model residuals indicate the differences between the observed values and the values predicted by the model. These values are symmetrically distributed around a median close to zero, indicating a good fit of the model to the data. The minimum and maximum residuals are −104.364 and 130.620, respectively, with the first and third quartiles being −21.241 and 13.454 and with a median of −5.497.

The regression coefficients show the impacts of each variable on the dependent variable. The intercept is −559.4071 and is statistically significant (*p* = 0.010754). The variable PTFE has a coefficient of 8.0785 and is highly statistically significant (*p* = 0.000816). The variable coke has a coefficient of 0.3735, but its effect is not statistically significant (*p* = 0.864548). The variable titanium dioxide has a coefficient of −2.1771 and is also insignificant (*p* = 0.811129). The variable kaolin has a coefficient of 23.0490 and is highly statistically significant (*p* < 0.001). The variable ultra-PTFE has a coefficient of −7.4229 but is not significant (*p* = 0.343971). The variable sodium chloride has a coefficient of 27.9755 and is highly statistically significant (*p* < 0.001). The variable graphite has a coefficient of −3.5091 and is not significant (*p* = 0.118261). The variable carbon fiber has a coefficient of 0.3001 and is also not significant (*p* = 0.889591).The R-squared value for the model is 0.80, which means that the model explains 80% of the variability in the relative strain of the composite. The variables PTFE, kaolin, and sodium chloride are highly statistically significant, while the other variables, such as coke, titanium dioxide, ultra-PTFE, graphite, and carbon fiber, do not have a significant effect on the relative elongation. But mostly, fillers reduce the relative elongation of PTFE (Figure 12c and Figure 13c), as they reduce the mobility of macromolecules and increase stiffness and resistance to deformation. The analysis of Figure 12c notes the atypical influence of kaolin and sodium chloride, which had little effect on the reduction of this indicator against the background of other fillers. So, the model considers them highly statistically significant.

Based on the results of the linear regression model for wear intensity, the model can be described as follows:

Wear intensity = −393.1092 + 4.5896⋅PTFE + 2.6485⋅Coke + 0.4709⋅Titanium_dioxide − 4.3168⋅Kaolin + 7.4668⋅Ultra_PTFE + 19.4094⋅Sodium_chloride + 2.5578⋅Graphite + 2.2832⋅Carbon_fiber(9)

The residuals of the model indicate the differences between the observed values and the values predicted by the model. These values are symmetrically distributed around a median close to zero, suggesting that the model fits the data well. The minimum and maximum residuals are −37.237 and 85.510, respectively, with the first and third quartiles being −11.818 and 8.586, and with a median of −0.727.

The regression coefficients show the impacts of individual variables on the dependent variable. The intercept value is −393.1092 and is statistically significant (*p* = 0.000198). The variable PTFE has a coefficient of 4.5896 and is highly statistically significant (*p* = 5.81 × 10^−5^). The variable coke has a coefficient of 2.6485 and is significant (*p* = 0.011256). The variable titanium dioxide has a coefficient of 0.4709 and is not significant (*p* = 0.909464). The variable kaolin has a coefficient of −4.3168 and is close to significance (*p* = 0.074956). The variable ultra-PTFE has a coefficient of 7.4668 and is significant (*p* = 0.041117). The variable sodium chloride has a coefficient of 19.4094 and is highly statistically significant (*p* = 5.41 × 10^−9^). The variable graphite has a coefficient of 2.5578 and is significant (*p* = 0.014762). The variable carbon fiber has a coefficient of 2.2832 and is significant (*p* = 0.025268).

The R-squared value of the model is 0.7949, which means that the model explains 79.49% of the variability in the composite’s wear intensity. This high value indicates a very good fit of the model to the data.

The presented model for wear intensity shows that the variables PTFE, coke, ultra-PTFE, sodium chloride, graphite, and carbon fiber have a significant effect on the wear intensity of the composite. The variables PTFE, sodium chloride, and ultra-PTFE are highly statistically significant, while Kaolin is close to significant. The variable titanium dioxide has no significant effect on the wear intensity.

To correctly interpret the wear rate prediction (Figure 12d and Figure 13d), we need to understand that the minimum values of this indicator provide the maximum wear resistance of the material, and vice versa. Therefore, for example, kaolin had the most significant effect on reducing the wear rate (Figure 12d), and the model does not consider this. On the other hand, the model has effectively noted the important influence of other significant fillers, such as solid lubricants (coke and graphite) and carbon fibers, which are traditionally added to the polymer to improve its tribological properties (Figure 13d). Nevertheless, for the correct interpretation of the wear prediction, it is necessary to consider additional analyses of the impact results, indicating the insufficient accuracy of this model.

Summarizing the models presented, we see that all models have high R-squared values, indicating a good fit to the data.

- PTFE is significant for relative elongation, wear intensity and density, but not for tensile strength.
- Kaolin has a significant effect on relative elongation and tensile strength, is close to significance for wear intensity, and is not significant for density.
- Sodium chloride is significant for relative elongation and wear intensity but not for density and tensile strength.
- Graphite is significant for density, wear intensity, and tensile strength, but not for relative elongation.
- Carbon fiber is significant for wear intensity and density but not for relative elongation and tensile strength.
- Titanium dioxide is not significant in any of the models.

These comparisons show that different variables have different effects on specific properties of the composite, which may be important when designing materials with specific mechanical and physical properties. In addition, for the final decision on the rational choice of the type and concentration of the filler, the optimum of the complex functional properties of the composite material must be considered. This is the optimal (sometimes sufficient, and not the highest, for example) value of each characteristic when reaching this condition with all indicators (density, strength, elongation, and wear intensity).

Despite the high efficiency of the proposed approach for predicting composite properties, several important limitations must be emphasized. First, the predictability of the results may be limited by the significant variability of the mechanical and physical properties within a given PTFE content range, especially between 90% and 98%, which may affect the reproducibility of the results. Secondly, the issue of multicollinearity between certain variables, particularly for basalt fibers, requires further analysis to improve the accuracy of the prediction models. In addition, some components, such as titanium dioxide, did not show a significant effect on composite properties, suggesting the need for further research on their role and influence under different application conditions. Another critical limitation is the lack of consideration of external factors such as temperature variations, long-term degradation, and environmental influences, which can significantly affect the durability and performance of the material in industrial applications. Another aspect requiring further optimization is a better understanding of the interactions between composite components at the microscopic level and the effects of their distribution on the macroscopic properties. Future research aims to extend the analyses with more advanced predictive models and experimental validation under real operating conditions.

## 6. Conclusions

This work investigated the effects of various fillers on the mechanical and tribological properties of PTFE-based composite materials, and developed a machine-learning approach for property prediction. The study has demonstrated that a rational selection of fillers and their concentrations allows for the development of tailored PTFE-based composites with enhanced performance for industrial applications.

The microstructural analysis confirmed that carbon and basalt fibers improved physical adhesion with the PTFE matrix, leading to a homogeneous distribution and reduced void formation. Kaolin and graphite formed intercalated structures that enhanced lubrication, while coke and ultra-PTFE contributed to improved self-lubrication under friction conditions. Sodium chloride exhibited weak interfacial adhesion, leading to unstable matrix interactions, while titanium dioxide nanoparticles achieved better dispersion due to high surface energy.

Optimal filler concentrations for PTFE-based composites were identified to balance mechanical properties and wear resistance, with 20 wt. % carbon fibers, 10 wt. % basalt fibers, 2 wt. % kaolin, 20 wt. % coke, 10 wt. % graphite, 5 wt. % sodium chloride, 3 wt. % titanium dioxide, and 1 wt. % ultra-dispersed PTFE providing the best performance. For three-component composites, the most effective combinations were 10% carbon and 10% basalt fibers, 14% carbon fibers and 6% kaolin, 5% carbon fibers and 15% coke, and 15% carbon fibers and 5% graphite, optimizing strength, durability, and wear resistance.

Regression models demonstrated high accuracy, explaining 74% of density, 67% of tensile strength, 80% of elongation, and 79% of wear intensity variations. PTFE significantly influenced elongation, wear intensity, and density, while kaolin and graphite impacted strength and wear properties. Sodium chloride affected elongation and wear, whereas titanium dioxide showed no significant effect. Despite the predictive efficiency of models, limitations include the mechanical variability in PTFE content (90–98%), multicollinearity, and unaccounted-for external factors affecting long-term material performance.

This study supports SDG 9 (Industry, Innovation, and Infrastructure) by integrating machine learning into material design, reducing experimental testing, minimizing material waste, and lowering production costs for industrial applications.

## Figures and Tables

**Table 1:** Main parameters of the polymer matrix.

Parameter	Value
Average particle size, μm	50–500
Density, kg/m^3^	2200
Tensile strength, MPa	23
Relative elongation at break, %	350

**Table 2:** Chemical composition (wt. %) of carbon fabric Carbon U-150.

C	H	O	B	P
60–65	1.1–4.5	3.5–4.5	3.0–3.6	3.0–3.6

**Table 3:** Chemical composition (wt. %) of basalt ultrafine fibers.

SiO_2_	Al_2_O_3_	FeO	Fe_2_O_3_	CaO	MgO	MnO	Na_2_O	TiO_2_
51.56	15.49	10.43	4.42	8.5	5.22	0.2	2.1	2.08

**Table 4:** Chemical composition (wt. %) of KS-1 brand kaolin.

SiO_2_	TiO_2_	Al_2_O_3_	Fe_2_O_3_	FeO	MgO	CaO	Na_2_O	K_2_O	H_2_O
45.81	0.72	39.24	0.13	–	0.31	0.52	–	–	0.13

**Table 5:** Characteristics of fillers.

Filler	Chemical Nature	Shape	Size, μm	Density, kg/m^3^
Fibrous				
Carbon fibers (CF)	synthetic inorganic	fibrous (cylindrical)	ø =10–12 l = 100–150	1510
Basalt fibers (BF)	synthetic inorganic	fibrous (cylindrical)	ø =2 l = 50–200	30–125
Dispersed				
Coke	natural organic	irregular	10–50	1730
Graphite	natural inorganic	lamellar (scaly)	15–30	1600
Kaolin	natural inorganic	lamellar (scaly)	up to 5	2580
Sodium chloride (NaCl)	synthetic inorganic	irregular	up to 600	2165
Nano				
Ultra PTFE (UPTFE)	synthetic organic	spherical	0.5–0.6	1900–2000
Titanium dioxide (TiO_2_)	synthetic inorganic	spherical	up to 0.4	3900–4250

**Table 6:** Designed two-component PTFE PCMs, wt. %.

No	PTFE	Carbon Fiber	Basalt Fiber	Coke	Graphite	Kaolin	Sodium Chloride	Ultra PTFE	Titanium Dioxide
1	90	10	−	−	−	−	−	−	−
2	85	15	−	−	−	−	−	−	−
3	80	20	−	−	−	−	−	−	−
4	75	25	−	−	−	−	−	−	−
5	97	−	3	−	−	−	−	−	−
6	92	−	8	−	−	−	−	−	−
7	90	−	10	−	−	−	−	−	−
8	88	−	12	−	−	−	−	−	−
9	85	−	15	−	−	−	−	−	−
10	95	−	−	5	−	−	−	−	−
11	90	−	−	10	−	−	−	−	−
12	85	−	−	15	−	−	−	−	−
13	80	−	−	20	−	−	−	−	−
14	95	−	−	−	5	−	−	−	−
15	90	−	−	−	10	−	−	−	−
16	85	−	−	−	15	−	−	−	−
17	80	−	−	−	20	−	−	−	−
18	98	−	−	−	−	2	−	−	−
19	96	−	−	−	−	4	−	−	−
20	94	−	−	−	−	6	−	−	−
21	98	−	−	−	−	−	2	−	−
22	95	−	−	−	−	−	5	−	−
23	92	−	−	−	−	−	8	−	−
24	99	−	−	−	−	−	−	1	−
25	98	−	−	−	−	−	−	2	−
26	97	−	−	−	−	−	−	3	−
26	96	−	−	−	−	−	−	4	−
27	95	−	−	−	−	−	−	5	−
28	99	−	−	−	−	−	−	−	1
29	97	−	−	−	−	−	−	−	3
30	95	−	−	−	−	−	−	−	5

**Table 7:** Designed three-component PTFE PCMs, wt. %.

No	PTFE	Carbon Fiber	Basalt Fiber	Coke	Graphite	Kaolin
31	80	5	15	−	−	−
32	80	10	10	−	−	−
33	80	15	5	−	−	−
34	75	20	5	−	−	−
35	80	18	−	−	−	2
36	80	16	−	−	−	4
37	80	14	−	−	−	6
38	80	15	−	5	−	−
39	80	10	−	10	−	−
40	80	5	−	15	−	−
41	80	15	−	−	5	−
42	80	10	−	−	10	−
43	80	5	−	−	15	−

## Data Availability

The original contributions presented in this study are included in the article. Further inquiries can be directed to the corresponding author.
